# An Epigenome-Wide DNA Methylation Map of Testis in Pigs for Study of Complex Traits

**DOI:** 10.3389/fgene.2019.00405

**Published:** 2019-04-30

**Authors:** Xiao Wang, Haja N. Kadarmideen

**Affiliations:** Quantitative Genomics, Bioinformatics and Computational Biology Group, Department of Applied Mathematics and Computer Science, Technical University of Denmark, Kongens Lyngby, Denmark

**Keywords:** pig, testis, epigenome, DNA methylation, RRBS, DMC

## Abstract

Epigenetic changes are important for understanding complex trait variation and inheritance in pigs that are also a valuable biomedical model for human health research. Testis is the main organ for reproduction and boar taint in pigs; however, there have been no studies to-date on adult pig testis epigenome. The main objective of this study was to establish a genome-wide DNA methylation map of pig testis that would help identify candidate epigenetic biomarkers and methylated genes for complex traits such as male reproduction, fertility or boar taint. Reduced Representation Bisulfite Sequencing (RRBS) was used to study methylation levels of cytosine in nine pig testis samples. The results showed that genome-wide methylation status of nine samples overlapped greatly and their variation among pigs were low. The methylation levels of promoter, exon, intron, cytosine and guanine dinucleotide (CpG) islands and CpG island shores regions were 0.15, 0.47, 0.55, 0.39, and 0.53, respectively. Cytosines binding to CpG islands showed different methylation levels between exon and intron regions. All methylation levels of CpG islands were lower than CpG island shores in different genic features. The distribution of 12,738 differentially methylated cytosines (DMCs) within CpG islands, CpG island shores and other regions was 36.86, 21.65, and 41.49%, respectively, and was 0.33, 1.71, 5.95, and 92.01% in promoter, exon, intron and intergenic regions, respectively. Methylation levels of DMCs in promoter, exon and intron regions were significantly different between CpG islands and CpG island shores (*P* < 0.05). A total of 898 genes with 2089 DMCs were enriched in 112 Gene Ontology (GO) terms. Fifteen methylated genes from our study were associated with fertility or boar taint traits. Our analysis revealed the methylation patterns in different genic features and CpG island regions of testis in pigs, and summarized several candidate genes associated with DMCs and the involved GO terms. These findings are helpful to understand the relationship between DNA methylation and genic CpG islands, to provide candidate epigenetic regions or biomarkers for pig production and welfare and for translational epigenomic studies that use pigs as an animal model for human research.

## Introduction

Pig is a valuable biomedical model of human obesity and metabolic diseases due to the anatomic, biochemical, pharmacological, pathological, and physiological similarities to the human (Kogelman et al., 2013; Kogelman and Kadarmideen, 2016). The previous study showed that the key role of epigenetic mechanisms in male gamete could widely affect human reproduction (Stuppia et al., 2015). Testis is the reproductive gland to produce sperm, so studying epigenetics of testis in pigs could improve our understanding of epigenetic molecular mechanisms related to male fertility and semen quality. Testis epigenome is also essential for the study of inheritance of boar taint in pigs – an unpleasant smell originating from cooking pork meat from uncastrated male pigs that is inherited (Strathe et al., 2013). Epigenetics is defined as changes in gene function that are heritable and no change in DNA sequence (Wu and Morris, 2001). As a major epigenetic modification, DNA methylation has been examined to be associated with growth (Jin et al., 2014), immune response (Wang et al., 2017), and reproduction traits (Bell et al., 2011) in pigs.

With high density of DNA methylation of cytosine and guanine dinucleotides (CpGs), CpG islands play an important role in gene regulation and transcriptional repression (Goldberg et al., 2007). The genome around the CpG islands can be widely affected by the methylation levels (Long et al., 2017). CpG island shores are strongly related to a specific tissue and are involved in modulating gene expression (Doi et al., 2009; Irizarry et al., 2009b). Most variable regions in terms of methylation such as methylation differences between tissues are CpG island shores rather than CpG islands themselves (Irizarry et al., 2009a; Hansen et al., 2011). DNA methylation in promoters is usually restricted to genes in a long-term stabilization of repressed states; therefore, promoter methylation can be a methylation inhibitor of therapeutic targets to silence genes (Yang et al., 2014). Most gene bodies are CpG-poor and extensively methylated, but their methylation can be a potential therapeutic target. Since DNA demethylation of the gene bodies could cause the down-regulation, so DNA methylation inhibitors can down regulate oncogenes and metabolic genes (Jones, 2012; Yang et al., 2014).

Reduced representation bisulfite sequencing (RRBS), based on next generation sequencing (NGS) technology, has been implemented to analyze patterns of DNA methylation by reducing the portion of the genome digestion (Meissner et al., 2005). Subsequently, reduced representation CpG sites are sequenced after restriction enzyme MspI digestion in CpG islands, promoters and enhancers (Smith et al., 2009). The RRBS method primarily focuses on the enrichment of CpG-rich regions rather than the non-CpG regions (Meissner et al., 2005). In mammals, DNA methylation almost exclusively occurs at CG dinucleotides with ratios of 70–80% throughout the genome (Ehrlich et al., 1982; Law and Jacobsen, 2010). Therefore, the information of CpG islands and gene-associated CpG sites can be provided by RRBS method (Choi et al., 2015). Currently, RRBS analysis of the pigs has been presented using intestinal tissue (Gao et al., 2014), ovaries (Yuan et al., 2016), and neocortex, liver, muscle and spleen (Choi et al., 2015).

Genome-wide DNA methylation patterns in porcine ovaries and porcine prepubertal testis have been profiled (Yuan et al., 2016; Chen et al., 2018), but to the best of our knowledge, genome-wide NGS-based methylation studies on adult testis epigenome in pigs have not been reported. The main objective of this study was to develop a map of DNA methylome for porcine testis using RRBS on nine testis samples of pigs and then characterize their methylome using bioinformatics methods. We characterized porcine adult testis epigenome by reporting the methylation levels and patterns in genic features and CpG islands for each testis sample. We identified differentially methylated cytosine (DMC) in nine sample to find DMC associated genes, and their involved Gene Ontology (GO) terms and pathways in pigs. Finally, we compared our results with other similar studies and provided a list of 15 candidate epigenetic biomarkers associated with male fertility (e.g., infertility, litter size, number of stillborn, and so on), boar taint (Skatole, Androstenone) and other complex traits linked to testis of pigs.

## Materials and Methods

### Pig Samples

Nine commercial purebred Landrace male pigs with similar genetic background from nine different sire families were raised by the same *ad libitum* feeding of same feed type in the same farm/environment. All pigs were slaughtered at an age of around 22 weeks by carbon dioxide (CO_2_) submersion at a commercial slaughterhouse (Danish Crown, Herning, Denmark), when they reached the slaughter weight of 105 kg. Testis tissue samples were retrieved by punch biopsy into the middle part of the testis with an inner punch distance of 2 cm. Thus, all of the testis samples were collected from the same part of the testis. Each sample weighed approximately 150 mg. These pigs were not treated by immunological castration or other castrating processes during the feeding period, so they had intact testis with normal fertility and viable sperms before or at slaughter.

Tissue samples were immediately immersed into the 1.5 ml RNAlater (QIAGEN, Hilden, Germany). All samples were stored at -20°C. Restriction enzyme digestion, adaptor ligation, size selection (40–220 bp fragments), bisulfite treatment, polymerase chain reaction (PCR) amplification and library construction were performed at BGI (Beijing Genomics Institute) Co., Ltd., Shenzhen, Guangdong, China. The nine samples were sequenced by a paired-end 100 bp flow cell in an Illumina HiSeq 2500 machine (PE-100bp FC; Illumina, San Diego, CA, United States) using RRBS method.

### Quality Control, Read Alignment, and Trimming

RRBS adapters and reads less than 20 bases long were trimmed by Trimmomatic software (version 0.36) (Bolger et al., 2014). Then, Bismark Bisulfite Mapper (version 0.19.0) (Krueger and Andrews, 2011) was applied to map clean reads to the porcine reference genome (Sscrofa11.1/susScr11) downloaded from the UCSC website^1^, and the cytosine methylation status was determined accordingly. Bismark Bisulfite Mapper includes three steps: genome preparation, alignment using Bowtie 2 (version 2.3.3.1) (Langmead and Salzberg, 2012) and methylation extractor. Bismark methylation extractor outputs read coverage and methylation percentage of detected methylated or unmethylated reads at one genomic position. The numbers of methylated and unmethylated CpG and non-CpG (CHG and CHH, H representing A/C/T) sites were also calculated for each sample. The read coverages lower than 10 counts were trimmed for discarding the unqualified reads. If an experiment suffered from PCR duplication bias, some clonal reads will impair accurate determination of methylation. Thus, cytosines with a percentile of read coverage higher than the 99.9th were also discarded for each sample.

### Genome-Wide DNA Methylation Levels and Methylation Patterns

The relationships of genome-wide methylation levels with densities of CpG islands, CpG island shores and genes were calculated through regression and correlation analysis, and counted by one mega base pairs (Mb) windows for each sample. Similarities and differences of genome structure, CpG islands and methylation level between genomic intervals were visualized by R package *RCircos* (version 1.2.0) (Zhang et al., 2013). Genic features were divided into promoter, exon and intron regions along the porcine genome. Afterward, we localized CpG islands and CpG island shores to these three genic features and investigated methylation patterns of genic CpG islands. Methylation patterns of CpG islands located at different genic features were visualized by R package *plot3D*.

### Differentially Methylated Cytosine (DMC) and Annotation

Methylation levels of cytosines were analyzed by the R package *methylKit* (version 1.4.0) (Akalin et al., 2012) based on the Bismark coverage file. Genome-wide cytosine sites were combined into one object to obtain the locations covered in all nine samples. In this study, methylation level of nine samples were considered as nine treatment levels in the logistic regression model to calculate *P*-values, which were then adjusted to *Q*-values using false discovery rate (FDR) to account for multiple hypothesis testing (Storey and Tibshirani, 2003). Chi-squared (χ^2^) test was used to determine the statistical significance of methylation differences between samples. Finally, we matched all DMCs into one file that included chromosomes, positions, *P*-values, *Q*-values, associated genes and their genic features, positions of CpG islands and CpG island shores and methylation levels of nine samples.

In this study, we defined CpG islands as a region with at least 200 bp, a GC fraction more than 0.5 and an observed-to-expected ratio of CpG more than 0.6. CpG island shores were then defined as regions of 2 kilo base pairs (kb) in length adjacent to CpG islands (Gardiner-Garden and Frommer, 1987). The CpG and DMC annotation within gene components of promoter, exon, intron and intergenic regions, and CpG islands, CpG island shores and other regions was performed using R package *genomation* (version 1.10.0) (Akalin et al., 2015). The porcine RefSeq and CpG island database (Sscrofa11.1/susScr11) for annotations were derived from the UCSC website^2^.

### Gene Ontology (GO) Enrichment and Pathway Analysis

GO enrichment and pathway analysis were analyzed in DAVID (Database for Annotation, Visualization and Integrated Discovery) Bioinformatics Resources 6.8^3^. NCBI reference sequences associated with DMCs were used in DAVID for the species of *Sus scrofa*. Significant GO terms and pathways were selected after filtering with *P* < 0.01. GO terms for the genes associated with DMCs were visualized by R package *GOplot* (version 1.0.2) (Walter et al., 2015).

## Results

### Statistics of Alignment With Porcine Reference Genome

In this study, bisulfite conversion efficiencies of these nine samples ranged from 98 to 99%. The RRBS sequencing generated approximately 59,328,166 read pairs per sample. On average, 58,604,646 read pairs survived the pre-processing step. The 49% of the remaining read pairs was uniquely aligned to the porcine reference genome. The reads pairs were located in 9,006,052 sites, which meant that the average depth of RRBS sequencing reads and uniquely aligned reads were approximately equal to 13 and 6.5, respectively (Table 1). A total of 871,462,976 averaged cytosines were analyzed from 28,944,768 uniquely aligned reads pairs including methylated and unmethylated cytosines in CpG/CHG/CHH contexts (Supplementary Table S1). It revealed that a paired-end 100 bp read evenly contained 30 analyzed cytosines. Additionally, a per-sample CpG methylation rate ranged from 46 to 53%. The per-sample average percentages of cytosine methylation rate in CHG and CHH sites were 0.89 and 0.63%, respectively (Table 1).

**Table 1:** Statistics of clean reads’ alignment with porcine reference genome (Sscrofa11.1/susScr11) and methylation rates in CpG, CHG, and CHH contexts.

Sample	Clean read pair	Uniquely aligned rate	Number of aligned site	Total number of analyzed cytosine	Cytosine methylation rate in CpG context	Cytosine methylation rate in CHG context	Cytosine methylation rate in CHH context
1	16,505,578	46%	6,555,417	210,492,580	49%	0.91%	0.61%
2	93,817,089	51%	11,786,693	1,458,034,594	53%	0.99%	0.69%
3	38,026,074	47%	8,350,750	507,968,318	46%	0.84%	0.58%
4	75,769,839	51%	11,024,632	1,161,664,236	52%	0.87%	0.62%
5	57,267,890	51%	10,230,855	994,282,472	50%	0.68%	0.52%
6	68,607,455	46%	8,427,406	881,065,710	46%	0.89%	0.64%
7	85,068,927	49%	8,799,356	1,220,798,901	49%	0.92%	0.67%
8	75,438,276	51%	9,259,657	1,194,394,820	51%	0.92%	0.67%
9	16,940,690	47%	6,619,706	214,465,154	50%	0.95%	0.66%
Mean	58,604,646	49%	9,006,052	871,462,976	50%	0.89%	0.63%
SD	28,617,798	2.3%	1,798,552	456,951,421	2.4%	0.09%	0.05%

**FIGURE 1:**
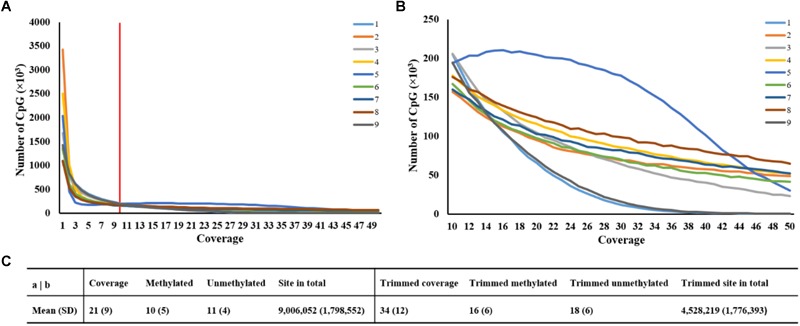
Statistics of averaged coverage in CpG context. **(A)** Number of CpG sites at different coverage of original data. Note: Red line indicated the coverage at 10. **(B)** Number of CpG sites at different coverage of trimmed data. **(C)** Comparison of statistics of averaged coverage between original and trimmed data.

It was obvious that the number of CpG sites was different at read coverage below 10, thus, the trimming criterion for read coverage was set at 10 (Figure 1A). Figure 1B revealed that the CpG site numbers of sample 1 and sample 9 were lower than the average value, while sample 5 has more CpG sites after trimming. Approximately, 9 million CpG sites were generated in each sample with read coverage equal to 21 (Figure 1C). After discarding coverage both lower than 10 and higher than 99.9th percentile, the averaged read coverage increased from 21 to 34, and the number of CpG sites reduced to a half (Figure 1C). The details of read coverages and methylation rates in CpG context of nine samples are listed in Supplementary Table S2. In addition, the coverage distributions per cytosine of nine samples after trimming are shown in Supplementary Figure S1. The percent methylation distributions per cytosine of nine samples after trimming were shown through histograms on the diagonal of Supplementary Figure S2.

### Genome-Wide DNA Methylation Status

**FIGURE 2:**
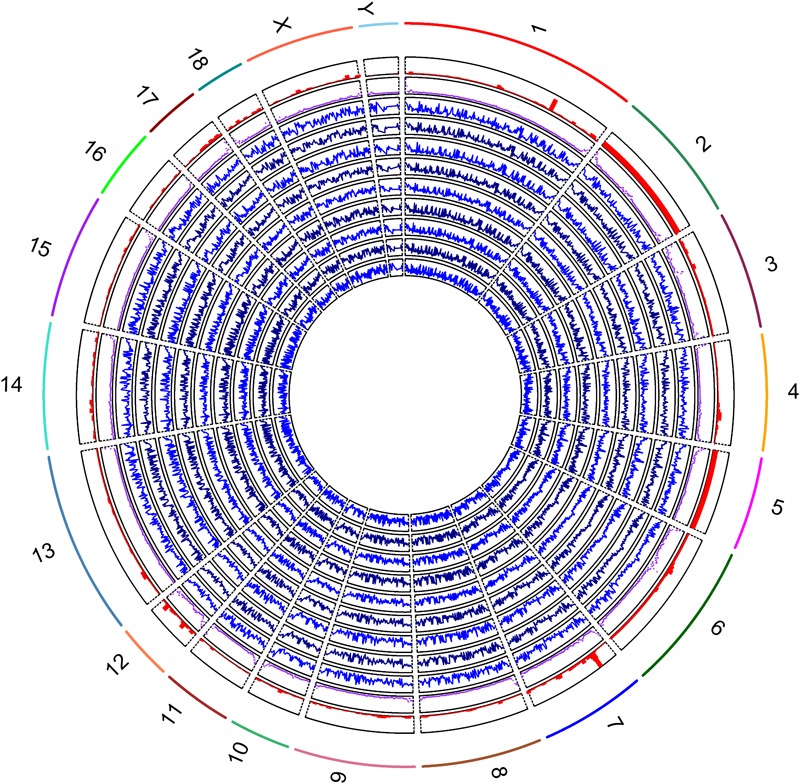
Global methylation levels of nine samples was shown by lines of in blue (track 1, 2, 3, 4, 5, 6 7, 8, and 9) from inside to outside. The methylation levels and the densities of CpG islands by scatter plot in purple color (track 10), and genes by histograms in red color (track 11) were counted by 1 Mb windows. The labels of outside track represented the chromosomes of the porcine genome.

The methylation levels against densities of CpG islands, CpG island shores and genes are shown in Figure 2. The genome-wide methylation status of nine samples showed the same trends and they overlapped greatly, suggesting that the biological variation between nine samples was low. Our analysis showed that the global CpG methylation rate was similar among the nine samples with Pearson’s correlation scores ranging from 0.95 to 0.98 (Supplementary Figure S2). The methylation levels varied across the different chromosomes with higher methylation variation in regions of low gene abundance, whereas lower methylation variation in those of high gene abundance (Figure 2). The regression coefficients of densities of genes, CpG islands and CpG island shores on methylation level were -2.20 (*P* < 0.001), 59.04 (*P* < 0.001), and 73.65 (*P* < 0.001), respectively, on average, over nine samples (Supplementary Figure S3 and Supplementary Table S3). The correlations between methylation levels and densities of genes, CpG islands and CpG island shores were -0.12, 0.25, and 0.23, respectively (Supplementary Table S3). These results suggested that genome hypomethylation in CpG islands was beneficial for the promotion of gene transcription, but their correlations were not so high.

### Methylation Patterns of CpG Islands Located at Different Genic Features

**FIGURE 3:**
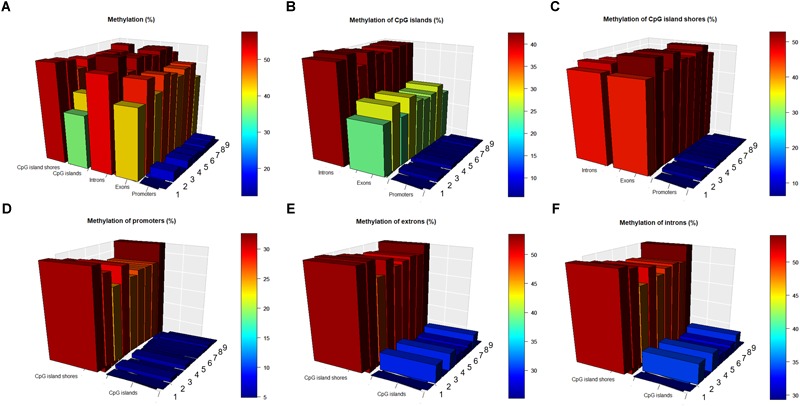
Methylation patterns in different genic features and CpG islands regions. **(A)** Methylation levels (in %) at different genic features, CpG islands and CpG island shores. **(B)** Methylation levels (in %) of CpG islands at different genic features. **(C)** Methylation levels (in %) of CpG island shores at different genic features. **(D)** Methylation levels (in %) of promoters in the CpG islands and CpG island shores. **(E)** Methylation levels (in %) of exons in the CpG islands and CpG island shores. **(F)** Methylation levels (in %) of introns in the CpG islands and CpG island shores.

To investigate the interaction of methylation levels between genes and CpG islands, we divided the porcine genome into three genic features (promoters, exons, and introns) and then localized CpG islands to these genic features. Methylation levels at different genic features and CpG islands displayed variously, with lowest values in the promoter regions. The methylation level were 0.15, 0.47, 0.55, 0.39, and 0.53 in the promoter, exon, intron, CpG islands, and CpG island shores regions, respectively, on average, over nine samples (Figure 3A). Comparisons of CpG islands and CpG island shores at different genic features revealed that the methylation levels of promoter regions were also the lowest. Meanwhile, CpG island shores located in intron regions showed slightly higher methylation levels than those located in exon regions, while CpG islands showed significant higher methylation levels (Figure 3B,C). Comparing with the methylation patterns in three different genic features, methylation levels of CpG islands were all lower than CpG island shores in the promoter, exon, and intron regions (Figure 3D–F).

### Differentially Methylated Cytosines (DMC) and Annotations

**FIGURE 4:**
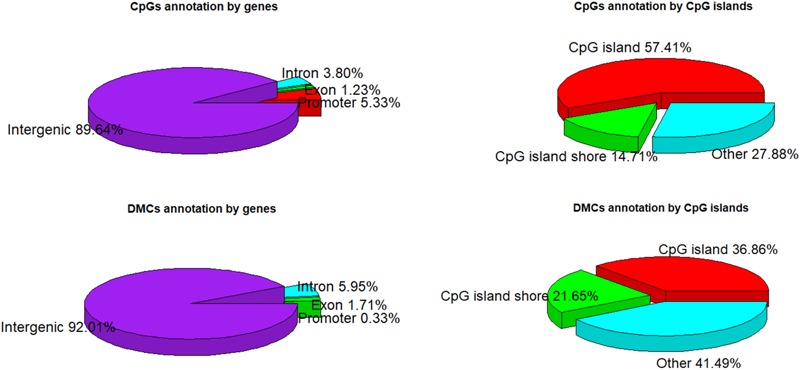
CpGs and DMCs annotation by genes and CpG islands.

A total of 1,244,043 CpG sites was covered in nine samples, and the number of identified DMCs was 12,738 with the level of *Q* < 0.01. Details of 12,738 DMCs with chromosomes, positions, *P*-values, *Q-*values, associated genes, genetic features and methylation levels are listed in Supplementary File S1. Percentages of 1,244,043 CpG sites annotated within promoter, exon, intron and intergenic regions were distributed as 5.33, 1.23, 3.80, and 89.64%, respectively. Additionally, the distribution of 1,244,043 CpG sites annotation within CpG islands, CpG island shores and other regions was 57.41, 14.71, and 27.88%, respectively. However, the distributions were 0.33, 1.71, 5.95, and 92.01% within promoter, exon, intron, and intergenic regions, respectively, when only considering the 12,738 DMCs. The distributions of DMCs annotated within CpG islands, CpG island shores and other regions were 36.86, 21.65, and 41.49%, respectively (Figure 4). The percentages of DMCs associated with CpG islands located in gene promoter, exon, intron, and intergenic regions were 69.05, 53.67, 32.32, and 36.72%, respectively. They were all higher than the DMCs associated with CpG island shores with the values of 19.05, 13.76, 24.01, and 21.66% in promoter, exon, intron, and intergenic regions, respectively (Table 2).

**Table 2:** DMCs associated with CpG island regions located at different genic features.

Genic feature	CpG island				CpG island shore			
	Promoter	Exon	Intron	Intergenic	Promoter	Exon	Intron	Intergenic
Number	29	117	245	4304	8	30	182	2538
Percentage	69.05%	53.67%	32.32%	36.72%	19.05%	13.76%	24.01%	21.66%

**FIGURE 5:**
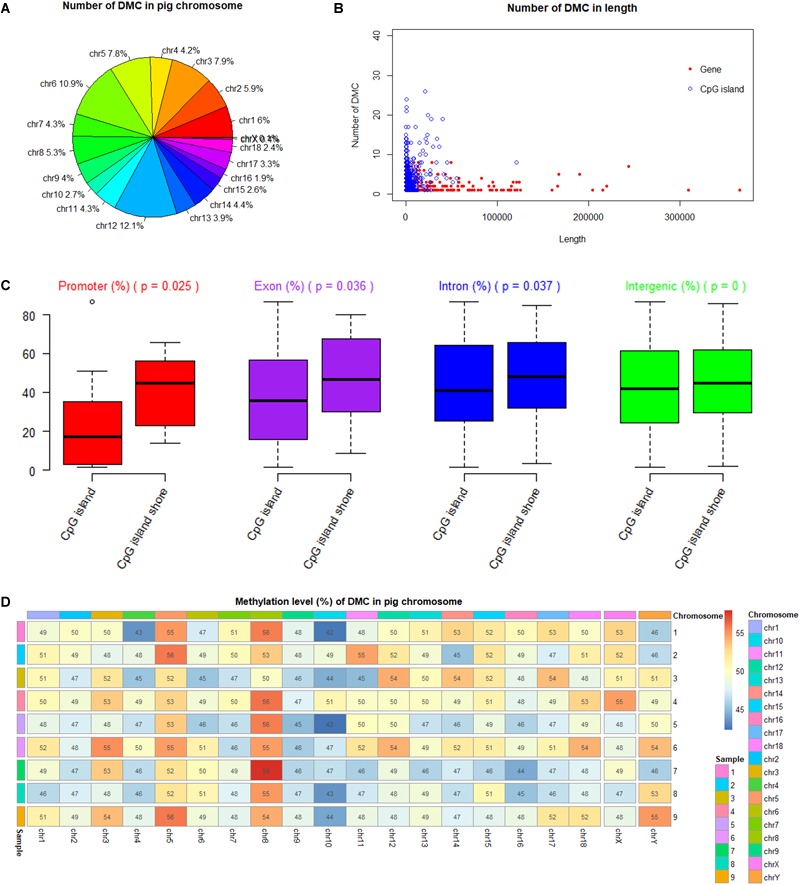
Methylation status of DMC in porcine chromosomes, genic features and CpG islands. **(A)** Number of DMC in different pig chromosome. **(B)** Number of DMC in the different lengths of genes and CpG islands. **(C)** Comparison of methylation levels between CpG islands and CpG island shores at different genic feature with Student’s *t*-tests. **(D)** Methylation levels of DMC in different pig chromosome.

Among 19 (*n* = 18 + 1) *Sus scrofa* chromosomes (SSC), DMCs occupied SSC12 (12.1%) mostly, and nearly no DMCs occupied SSC X and SSC Y with the percentages of 0.4 and 0.1%, respectively (Figure 5A). DMCs were located mostly in the shorter genes and to lesser extent in the longer genes. Similarly, most of DMCs were located in CpG islands with a short length from 200 to 1000 bp (Figure 5B). Methylation levels of DMCs in different genic features were different, with the lowest values of CpG islands in the promoter regions. Student’s *t*-tests showed that methylation levels of DMCs in promoter, exon and intron regions were significantly different between CpG islands and CpG island shores (*P* < 0.05), while those of intergenic regions were extremely significant (*P* < 0.001) (Figure 5C). The averaged methylation levels on different chromosomes and different individuals were similar, with values close to 50% (Figure 5D).

### Genes Associated With DMCs and Their Gene Ontology (GO) Enrichment and Pathway Analyses

We found that 976 DMCs were annotated within gene components of 415 genes after matching 12,738 DMCs to the porcine RefSeq database (Sscrofa11.1/susScr11) (Supplementary File S1). Fifteen genes associated with DMCs found to be related to fertility or boar taint traits were also reported by other studies (Table 3). Genes *ACACA, CYP21A2, CYP27A1, HSD17B2, LHB, PARVG*, and *SERPINC1* were associated with boar taint, while genes *DICER1, PCK1, SS18*, and *TGFB3* were associated with pig reproduction traits. In addition, the other five genes (*CAPN10, FTO, HSD17B2, IGF2*, and *SALL4*) were found to be associated with fertility traits in human, in which *HSD17B2* also played a role in boar taint (Table 3).

**Table 3:** Comparisons between DMC related genes of this study and identified genes for fertility or boat taint traits of other studies.

	This study				Other study				
Co-found gene	Chromosome	Number of DMC	DMC position	Feature region	Specie	Tissue	Data type	Associated trait	References
*ACACA*	SSC12	1	38585187	Intron	Pig	Testis	RNA-Seq	Androstenone	Moe et al., 2007
*CAPN10*	SSC15	1	139564776	Exon	Human	Leukocytes	SNP	Polycystic ovarian syndrome	Gonzalez et al., 2003
*CYP21A2*	SSC7	1	24086506	Intron and CpG island shore	Pig	Blood, leukocytes or semen	SNP array	Androstenone, testosterone, 17β-estradiol and estronsulphate.	Grindflek et al., 2011
*CYP27A1*	SSC15	1	120810825	Intron and CpG island	Pig	Testis	RNA-Seq	Boar taint	Drag et al., 2017
*DICER1*	SSC7	2	116375790, 116375791	Intron and CpG island shore	Pig	Testis and oviduct	RNA-Seq	Reproduction	Fischer et al., 2015
*FTO*	SSC6	2	31317922, 31317957	Intron	Human	Blood	SNP array	Polycystic ovarian syndrome	Barber et al., 2008
*HSD17B2*	SSC6	1	6296641	Exon	Pig/Human	Liver/ Endometrium	RNA-Seq /RNA	Skatole metabolism /Endometriosis	Zeitoun et al., 1998; Gunawan et al., 2013
*IGF2*	SSC2	1	1486537	Intron and CpG island shore	Human	Semen	DNA methylation	Male infertility	Poplinski et al., 2010
*LHB*	SSC6	1	54264270	Exon and CpG island shore	Pig		SNP array	Androstenone metabolism	Duijvesteijn et al., 2010
*PARVG*	SSC5	4	4874783, 4874934, 4874946, 4886005	Intron	Pig	Testis	RNA-Seq	Androstenone	Moe et al., 2007
*PCK1*	SSC17	1	57933961	Exon and CpG island shore	Pig	Placenta	RNA-Seq	litter size	Kwon et al., 2016
*SALL4*	SSC17	3	53089645, 53089693, 53090863	Exon and CpG island	Human	Peripheral blood leukocytes	SNP	Premature Ovarian Failure	Wang et al., 2009
*SERPINC1*	SSC9	1	116186998	Exon	Pig	Liver	RNA-Seq	Boar taint	Drag et al., 2017
*SS18*	SSC6	1	110725565	Intron	Pig	Ear	Whole genome	Number of stillborn	Onteru et al., 2012
*TGFB3*	SSC7	1	99138369	Exon	Pig		SNP array	Number of teats	Verardo et al., 2016

**FIGURE 6:**
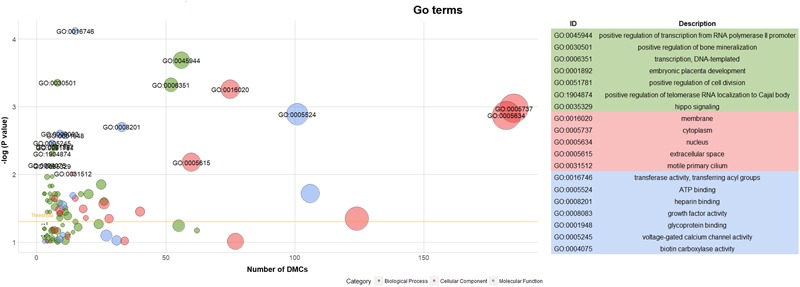
Go term analysis of genes associated with DMCs. Note: Yellow line in the left panel meant the threshold of significant GO terms (*P* < 0.05).

Hereafter, 898 genes (296 unique genes) associated with 2089 DMCs (704 unique DMCs) were enriched in 112 GO terms (Supplementary File S2). The significant GO terms (*P* < 0.01) are shown with the texts including 7 GO terms of biological process, 5 GO terms of cellular component and 7 GO terms of molecular function (Figure 6). Generally, as more genes were enriched in the GO terms, the number of included DMCs increased (Figure 6). Two GO terms (GO: 0005737 and GO: 0005634) in the cellular component contained the genes and DMCs mostly, that were 80 and 78 enriched genes associated with 185 and 182 DMCs, respectively (Supplementary File S2). The 23 significant pathways (*P* < 0.01) are listed in Supplementary Table S4. The most significant pathway was insulin signaling pathway (*P* = 9.89 × 10^-7^) containing 16 genes namely *PHKG2, FASN, PHKG1, ACACA, IKBKB, FBP1, GYS1, PRKCZ, PRKAA2, PRKAG1, PCK1, ACACB, PIK3R5, SREBF1, AKT2*, and *MAP2K1* (Supplementary Table S4).

## Discussion

Generally, the bisulfite conversion rates ranged from 90 to 100%, but some conversion rates varied between 99 and 100% depending on the commercial methods (Worm Ørntoft et al., 2017). This study showed higher bisulfite conversion efficiencies between 98 and 99%. A mapping efficiency of 38.3% was previously reported in RRBS sequencing of lamb muscle with fragment sizes of 50–150 bp, which increased to 61.4% with fragment sizes of 150–250 bp (Doherty and Couldrey, 2014). Similarly, our study revealed efficiency of 49% using 40–220 bp sizes that were uniquely mapped to the porcine reference genome (Table 1). It is consistent with 60% mapping rates using 110–220 bp sizes in RRBS sequencing for porcine ovaries (Yuan et al., 2016). We found that global CpG methylation levels ranged from 45 to 53% (50% on average), which is similar with other studies on pig methylation research using RRBS method (Gao et al., 2014; Choi et al., 2015; Schachtschneider et al., 2015), whereas non-CpG methylation levels (CHG and CHH sites) were less than 1% (Table 1). This is reasonable because CpGs within poor-CpG regions are scarcely covered based on restriction enzyme digestion by the RRBS method (Meissner et al., 2005). Our results also showed 72% of CpG methylations were mapped to CpG islands (57.41%) and to CpG island shores (14.71%), that were higher than those of Choi’s study (Choi et al., 2015). Whole genome bisulfite sequencing (WGBS) technology can produce many reads in poorly assembled non-coding DNA regions, resulting in lower mapping efficiency than RRBS method (Doherty and Couldrey, 2014). However, RRBS data sets have a somewhat lower average methylation level than WGBS data sets, because large stretches of repeat regions in non-coding DNA regions are generally highly methylated (Bird, 2002). Practically, some CpG sites had low coverage (1∼ 10) or are not even sequenced by the WGBS method, although all sites should be theoretically covered (Sun et al., 2015). Thus, average read depths of RRBS sequencing were higher than 10 in this study (Table 1 and Supplementary Table S1) and in other studies (Zhao et al., 2016; Carmona et al., 2017). Overall, RRBS method remained a better choice when considering sequencing cost, read coverage and sufficient methylation information (Choi et al., 2015).

In many cell types of different species, percentages of methylations would have a bimodal distribution, which denoted that the majority of bases has either high or low methylation to indicate a site specificity (Ehrlich et al., 1982). This bimodal pattern was a possible function to keep the factor-mediated basal transcription profile of the preimplantation embryo (Cedar and Bergman, 2012). The CpG methylation percentage distribution would be measured with two peaks at 0 and 100%, when a large number of the CpG sites were sequenced in either unmethylated or fully methylated status (Falckenhayn et al., 2013; Zhang et al., 2017). Bimodal distribution is also an important metric to help reveal whether the experiments suffer from PCR duplication bias. If there is a high degree of clonal reads from PCR, some reads will be asymmetrically amplified and read coverage distribution will have a secondary peak correspondingly on the right side. This situation will impair accurate determination of percent methylation scores for those regions. Hence, this study discarded cytosines with a percentile of read coverage higher than 99.9th, and then showed the reasonably bimodal distribution (Supplementary Figure 2) in consistency with other results using different tissues in pigs (Choi et al., 2015).

Not only did DNA methylation have a correlation with gene transcription, but also the presence of methyl moieties inhibited gene expression *in vivo* (Razin and Cedar, 1991). It was suggested by our study that the regression coefficients and correlation coefficients of genes and methylation levels were both negative, ranging from -1.97 to -2.46 and from -0.10 to -0.14, respectively (Supplementary Table S3). In practice, the correlation coefficient between gene expression and methylation level was approximately 0.3, negative (Bock, 2012). Methylated genes might be associated with genomic region-specific DNA methylation patterns (Raza et al., 2017), and therefore, this study investigated promoter, exon and intron regions along the porcine genome and localized CpG islands to these genic features. The interactions of methylations between three genic features and CpG islands suggested that methylation levels of promoter regions were lowest in both CpG islands and CpG island shores (Figure 3A). It was well known that DNA methylation in a promoter was correlated with the transcription of a target gene (Niesen et al., 2005). Methylation levels of CpG islands were lower than CpG island shores in the promoter, exon and intron regions in this study (Figure 3D–F). These results demonstrated that CpG islands located in different genic features displayed effects on the methylation patterns of the associated genes. Irizarry et al. (Irizarry et al., 2009b) revealed a strong relation between methylations in CpG island shores located within 2 kb of an annotated transcription start site (TSS) and expression of associated genes. Meanwhile, CpG islands located in exon regions showed different methylation level with those located in intron regions (Figure 3B,C), which suggested that exons had an effect on the methylation patterns of CpG islands. Chen et al. (2018) has profiled methylation patterns for porcine testis at three prepubertal age points (i.e., 1, 2, and 3 months). They found that the methylation levels of promoters and CpG islands decreased as the pig gradually matured, while methylation levels of gene body kept stable (Chen et al., 2018). It was suggested that lower methylations in promoters could be a specific pattern for testis tissue in adult pig, because spermatogenic cells tended to be activated for the increasing gene expression requirement at this stage. Additionally, Yuan et al. (2017) revealed that CpG islands show lower methylation levels compared to their CpG island shore regions in porcine hypothalamus-pituitary-ovary axis. Methylation levels in introns, exons, and promoters gradually decreased both in CpG islands and CpG island shores (Yuan et al., 2017). The methylation patterns of hypothalamus-pituitary-ovary axis were similar to our results except that exons located in CpG island shores of this study showed slightly higher methylations than those located in CpG islands (Figure 3B,C).

The percentages of DMCs annotation within exon, intron and intergenic regions increased, whereas DMCs annotation within promoter region decreased dramatically, when comparing DMCs with CpGs annotation within genic features. Similarly, the percentage of DMCs annotation within CpG island shores increased, while DMCs annotation within CpG islands decreased (Figure 4). As Maunakea et al. (2010) found that the methylated CpG islands in 5′ promoter regions were less than 3%, DMCs found in promoter regions were also less than 1% in this study (Figure 4). The most common promoter type in the vertebrate genome was annotated gene promoters with the CpG islands and they occupied at above 70% (Saxonov et al., 2006). We found that approximately 69% of DMCs associated with CpG islands were located in promoter regions (Table 2). Liu et al. (Liu et al., 2017) reported that the proportions of hypermethylated CpG sites located in CpG islands, CpG shores and other locations were 25.49∼34.23%, 21.57∼40.75%, and 25.02∼52.94%, respectively, during different stages of human embryonic stem cells. Genes that contained differentially methylated regions (DMRs) in their first intron were more than the genes that contained DMRs in their promoter and their first exon (Anastasiadi et al., 2018), which are the same trend as this study (Supplementary File S1).

In humans, more than 80% of sperm cells were mainly composed in the testis (Bellve et al., 1977). The epigenetic modifications of germ cells occurring in the meiotic and post-meiotic phases of spermatogenesis are crucial for embryonic development after fertilization (Marques et al., 2010). Due to the failure of re-methylation in spermatogonia or alterations to methylation maintenance in spermatocytes, sperm cells or the mature sperm cells, the abnormal DNA methylation patterns were observed in the infertile men (Cui et al., 2016). Therefore, the methylation patterns in genic features and CpG islands of pig testis were investigated to reveal significant cytosines and associated genes for epigenetic molecular mechanisms related to male fertility. Langenstroth-Röwer et al. (2017) used the marmoset monkey as the human model for testicular methylation study. They found that cytosines were predominantly unmethylated at regulatory regions of *H19, LIT1, SNRPN, MEST*, and *OCT4* in the germ cells. Meanwhile, DNA methylation pattern of *H19, MEST, DDX-4*, and *MAGE-A4* did not change in germ cell fractions (Langenstroth-Röwer et al., 2017). The genome-wide promoter methylation profiles identified 367 testis and epididymis-specific hypomethylated genes and 134 hypermethylated genes, many of them were involved in the GO terms of male reproduction (Wu et al., 2013). Compared with the fertile males, it was reported that a low methylation or unmethylation pattern at the *H19* was associated with hypermethylation at the *MEST* and a reduced sperm quality in the oligospermic patients (Niemitz and Feinberg, 2004). DMRs located in the upstream of TSS of the *H19* harbored several CCCTC-binding factor (CTCF) binding sites (Takai, 2001). However, CTCF binding to the maternal unmethylated DMR could prevent *IGF2* from accessing the common enhancers, and thus silencing its expression (Marques et al., 2010). Rajender et al. (2011) summarized that genes *MTHFR, PAX8, NTF3, SFN, HRAS, JHM2DA, IGF2, H19, RASGRF1, GTL2, PLAG1, D1RAS3, MEST, KCNQ1, LIT1*, and *SNRPN* were associated with male infertility. Our study also identified the DMCs located in the intron regions of *IGF2* (Table 3), which was involved in GO terms of positive regulation of cell division (GO: 0051781), extracellular space (GO: 0005615), and growth factor activity (GO: 0008083) (Supplementary File S2).

Our study revealed the methylation patterns in different genic features such as promotor, exon, intron and intergenic regions, as well as CpG islands, CpG island shores regions. Furthermore, our study reported many candidate genes harboring DMCs and the involved GO terms of testis in pig. Until now, several studies have concluded the important genes associated with male fertilities using SNP array, RNA-Seq datasets for humans (Table 3), however, epigenetic studies in pigs relating to male fertility are rare. This study has reported for the first time, DNA methylome (epigenomic) architecture in adult pig testis for study of male fertility in pigs. These results will also be useful for the study of boar taint in pigs associated with sensory meat quality, as boar taint is inherited and shows complex gene regulation patterns (Strathe et al., 2013; Drag et al., 2018). Since this study is based on sequence-level resolution of transmittable epigenetic changes, we believe it may also contribute to understanding and capturing part of the genetic variation that are not captured by SNP arrays (considered missing or “missing heritability”) in genome-wide genomic prediction studies. As pig is a valuable biomedical model of human, the findings of this study are also very helpful to understand the relationship between DNA methylation and genic CpG islands, and provide candidate epigenetic biomarkers for the translational studies in human research.

## Conclusion

This is the first study to report catalog of adult pig testis epigenome by developing a genome-wide DNA methylation map with the use of RRBS technology. We found that the methylation rates were lowest in promoters (0.15) and highest in introns (0.55). Cytosines binding to CpG islands showed different methylation patterns between intron and exon regions. Methylation levels of CpG islands were lower than CpG island shores in different genic features. We detected 12,738 DMCs in total. They distributions of DMCs within CpG islands, CpG island shores and other regions were 36.86, 21.65, and 41.49%, respectively. The distributions of DMCs were 0.33, 1.71, 5.95, and 92.01% in promoter, exon, intron and intergenic regions, respectively. Fifteen genes with DMCs were associated with human fertility (*ACACA, CYP21A2, CYP27A1, HSD17B2, LHB, PARVG*, and *SERPINC1*), pig reproduction (*DICER1, PCK1, SS18*, and *TGFB3*) and boar taint traits (*CAPN10, FTO, HSD17B2, IGF2*, and *SALL4*). These findings on genome-wide epigenetic signatures will be useful to understand testis-related trait inheritance in pigs (e.g., male fertility, semen quality, boar taint) for pig production and welfare. This study, based on sequence-level resolution of epigenetic changes, also contributes to understanding and capturing part of the genetic variation that are considered missing (“missing heritability”) in genome-wide genomic prediction studies. Since pigs are useful as an animal model for human research, epigenetic architecture of pigs would help in translational research.

## Ethics Statement

Animal Care and Use Committee approval was not obtained for this study, because tissue samples were obtained from a commercial slaughter facility.

## Author Contributions

HK conceived, designed and implemented the epigenomic experiments including collection of tissue samples and processing of samples for methylome sequencing by RRBS, and improved the manuscript. XW analyzed the data. XW and HK interpreted the results and wrote the manuscript. Both authors read and approved the final manuscript.

## Conflict of Interest Statement

The authors declare that the research was conducted in the absence of any commercial or financial relationships that could be construed as a potential conflict of interest.

## Footnotes

1 http://hgdownload.cse.ucsc.edu/goldenPath/susScr11/bigZips/susScr11.fa.gz

2 http://genome.ucsc.edu/cgi-bin/hgTables

3 https://david.ncifcrf.gov/

## Supplementary Material

FIGURE S1 Histograms of log10 of read coverage per CpG site.

FIGURE S2 Correlation analysis of the global CpG methylation patterns among nine samples. Note: Colors in the scatter plot indicate the number of CpG sites with identical methylation pattern (methylated or non-methylated): yellow denotes many correlations, blue denotes lack of correlation and green denotes different methylation patterns. Numbers in the upper right side represent the pairwise Pearson’s correlation scores. Histograms on the diagonal are methylation distribution per CpG site for each sample.

FIGURE S3 Regression of densities of genes, CpG islands and CpG island shores on methylation levels from one sample, all counted by 1 Mb windows.

TABLE S1 Total number of aligned cytosine methylation in different contexts.

TABLE S2 Statistics of coverage and methylation rates in CpG context.

TABLE S3 Regression and correlation analysis of densities of genes, CpG islands and CpG island shores on methylation levels, all counted by 1 Mb windows.

TABLE S4 Significant pathways (*P* < 0.01).

FILE S1 Details of 12,738 DMCs with chromosomes, positions, *P*-values, *Q*-values, associated genes, genetic features and methylation levels.

FILE S2 GO terms and enriched genes associated with DMCs.

## Abbreviations

BGI: Beijing Genomics Institute
bp: base pair
cm: centimetre
CO2: Carbon dioxide
CpG: Cytosine and guanine dinucleotide
CTCF: CCCTC-binding factor
DMC: Differentially methylated cytosine
DMR: Differentially methylated region
FDR: False discovery rate
GO: Gene ontology
kb: kilo base pairs
kg: kilogram
Mb: mega base pair
mg: milligram
ml: millilitre
NGS: Next generation sequencing
PCR: Polymerase chain reaction
RNA-Seq: RNA sequencing
RRBS: Reduced representation bisulfite sequencing
SNP: Single nucleotide polymorphism
SSC: *Sus scrofa* chromosomes
TSS: Transcription start site
WGBS: Whole genome bisulfite sequencing
