# Supplementary material for: An Epigenome-Wide DNA Methylation Map of Testis in Pigs for Study of Complex Traits
Source: Front Genet. 2019 Apr 30;10:405. doi: 10.3389/fgene.2019.00405 (PMC6502962; doi:10.3389/fgene.2019.00405)
Supplement: FIGURE S1 — Histograms of log10 of read coverage per CpG site. [file Data_Sheet_3.docx]

**Genome-wide DNA methylation map of testis in pigs for study of complex trait in epigenetics**

Xiao Wang^1,2^, Haja N. Kadarmideen^1,2*^

*^1^Quantitative Genomics, Bioinformatics and Computational Biology Group, Department of Bio and Health Informatics and ^2^Department of Applied Mathematics and Computer Science, Technical University of Denmark, Richard Petersens Plads, Building 324, 2800, Kongens Lyngby, Denmark*

Supplementary table 1. Total number of aligned cytosine methylation in different contexts.

| Sample | Raw read pair | Removal of RRBS adapters pollution | Removal of reads less than 20 bases long pollution | Uniquely aligned read pair | Total methylated cytosine in CpG context | Total methylated cytosine in CHG context | Total methylated cytosine in CHH context | Total unmethylated cytosine in CpG context | Total unmethylated cytosine in CHG context | Total unmethylated cytosine in CHH context |
| --- | --- | --- | --- | --- | --- | --- | --- | --- | --- | --- |
| 1 | 16,698,176 | 16,676,459 | 16,505,578 | 7,626,224 | 25,285,774 | 533,874 | 613,446 | 25,954,263 | 58,429,002 | 99,676,221 |
| 2 | 95,226,092 | 94,982,628 | 93,817,089 | 47,664,062 | 176,804,459 | 4,035,546 | 4,935,839 | 157,691,156 | 403,645,743 | 710,921,851 |
| 3 | 38,428,786 | 38,398,933 | 38,026,074 | 17,805,056 | 56,793,321 | 1,183,759 | 1,405,332 | 67,690,550 | 139,729,393 | 241,165,963 |
| 4 | 76,547,112 | 76,492,906 | 75,769,839 | 38,737,948 | 138,976,823 | 2,832,374 | 3,546,545 | 126,425,365 | 322,401,427 | 567,481,702 |
| 5 | 58,288,942 | 58,203,032 | 57,267,890 | 29,233,916 | 101,157,611 | 1,824,688 | 2,734,279 | 99,455,692 | 265,705,802 | 523,404,400 |
| 6 | 69,409,837 | 69,277,350 | 68,607,455 | 31,560,762 | 99,474,166 | 2,177,276 | 2,680,962 | 116,309,843 | 241,862,604 | 418,560,859 |
| 7 | 85,946,536 | 85,848,515 | 85,068,927 | 41,686,025 | 139,527,755 | 3,118,644 | 3,983,459 | 143,670,905 | 336,163,467 | 594,334,671 |
| 8 | 76,287,408 | 76,091,969 | 75,438,276 | 38,315,536 | 134,584,159 | 3,001,451 | 4,062,527 | 128,653,677 | 324,817,894 | 599,275,112 |
| 9 | 17,120,606 | 17,100,153 | 16,940,690 | 7,873,386 | 26,155,846 | 568,832 | 672,578 | 26,163,882 | 59,605,482 | 101,298,534 |
| Mean | 59,328,166 | 59,230,216 | 58,604,646 | 28,944,768 | 99,862,213 | 2,141,827 | 2,737,219 | 99,112,815 | 239,151,202 | 428,457,701 |
| SD | 28,981,902 | 28,919,705 | 28,617,798 | 14,688,316 | 53,712,269 | 1,216,332 | 1,555,183 | 48,724,247 | 125,615,280 | 227,796,188 |

Supplementary table 2. Statistics of coverage and methylation rates in CpG context.

| Sample | 1 | 2 | 3 | 4 | 5 | 6 | 7 | 8 | 9 | Mean | SD |
| --- | --- | --- | --- | --- | --- | --- | --- | --- | --- | --- | --- |
| Coverage | 7.82 | 28.38 | 14.91 | 24.07 | 19.61 | 25.61 | 32.18 | 28.43 | 7.90 | 20.99 | 9.01 |
| Methylated | 3.86 | 15.00 | 6.80 | 12.61 | 9.89 | 11.80 | 15.86 | 14.53 | 3.95 | 10.48 | 4.64 |
| Unmethylated | 3.96 | 13.38 | 8.11 | 11.47 | 9.72 | 13.80 | 16.33 | 13.89 | 3.95 | 10.51 | 4.44 |
| Methylated rate (%) | 49.35% | 52.85% | 45.61% | 52.39% | 50.43% | 46.08% | 49.29% | 51.11% | 50.01% | 49.92% | 51.51% |
| Site in total | 6,555,417 | 11,786,693 | 8,350,750 | 11,024,632 | 10,230,855 | 8,427,406 | 8,799,356 | 9,259,657 | 6,619,706 | 9,006,052 | 1,798,552 |
| Trimmed coverage | 16.52 | 50.31 | 26.71 | 40.83 | 27.13 | 40.20 | 44.95 | 38.83 | 17.10 | 33.62 | 12.17 |
| Trimmed methylated | 7.34 | 25.57 | 11.01 | 20.59 | 13.46 | 17.83 | 21.76 | 19.26 | 7.86 | 16.08 | 6.45 |
| Trimmed unmethylated | 9.19 | 24.74 | 15.70 | 20.24 | 13.67 | 22.37 | 23.19 | 19.58 | 9.24 | 17.55 | 5.87 |
| Trimmed methylated rate (%) | 44.41% | 50.82% | 41.22% | 50.43% | 49.61% | 44.35% | 48.41% | 49.60% | 45.98% | 47.20% | 3.35% |
| Trimmed site in total | 1,740,792 | 5,716,743 | 3,517,675 | 5,490,560 | 6,415,652 | 4,577,586 | 5,559,731 | 5,942,865 | 1,792,367 | 4,528,219 | 1,776,393 |

Supplementary table 3. Regression and correlation analysis of densities of genes, CpG islands and CpG island shores on methylation levels, all counted by 1 Mb windows.

| Sample ID | Gene | | CpG island | | CpG island shore | |
| --- | --- | --- | --- | --- | --- | --- |
|  | Regression coefficient | Correlation coefficient | Regression coefficient | Correlation coefficient | Regression coefficient | Correlation coefficient |
| 1 | -2.22 | -0.13 | 44.84 | 0.21 | 53.07 | 0.18 |
| 2 | -2.03 | -0.12 | 59.09 | 0.26 | 74.61 | 0.24 |
| 3 | -2.09 | -0.12 | 58.49 | 0.27 | 72.99 | 0.24 |
| 4 | -1.97 | -0.10 | 63.64 | 0.26 | 80.71 | 0.24 |
| 5 | -2.21 | -0.11 | 74.26 | 0.28 | 95.72 | 0.27 |
| 6 | -2.33 | -0.13 | 56.34 | 0.25 | 69.24 | 0.22 |
| 7 | -2.04 | -0.11 | 71.31 | 0.29 | 90.87 | 0.27 |
| 8 | -2.44 | -0.13 | 60.32 | 0.25 | 75.58 | 0.23 |
| 9 | -2.46 | -0.14 | 43.04 | 0.19 | 50.06 | 0.16 |

Supplementary table 4. Significant pathways (P < 0.01).

| Term | Gene | Count | % | P value | Fold Enrichment | Bonferroni | Benjamini | FDR |
| --- | --- | --- | --- | --- | --- | --- | --- | --- |
| ssc04910:Insulin signaling pathway | *PHKG2, FASN, PHKG1, ACACA, IKBKB, FBP1, GYS1, PRKCZ, PRKAA2, PRKAG1, PCK1, ACACB, PIK3R5, SREBF1, AKT2, MAP2K1* | 16 | 5.41 | 9.89E-7 | 4.77 | 2.24E-4 | 2.24E-4 | 0.00127 |
| ssc04152:AMPK signaling pathway | *FASN, ACACA, FBP1, GYS1, ADRA1A, PPP2R2B, CPT1B, CPT1A, PRKAA2, PRKAG1, PCK1, FOXO3, PIK3R5, SREBF1, AKT2* | 15 | 5.07 | 1.35E-6 | 5.01 | 3.06E-4 | 1.53E-4 | 0.00173 |
| ssc04390:Hippo signaling pathway | *GLI2, BMP7, BMP6, SOX2, TEAD3, BMPR1B, TEAD4, TEAD1, PRKCZ, PPP2R2B, TGFB1, LATS2, ITGB2, TGFB3, CCND2, WNT4* | 16 | 5.41 | 1.76E-6 | 4.56 | 3.99E-4 | 1.33E-4 | 0.00225 |
| ssc04931:Insulin resistance | *IKBKB, GYS1, PRKCZ, NOS3, CPT1B, PRKAG1, PRKAA2, CPT1A, PCK1, ACACB, PIK3R5, SREBF1, AKT2* | 13 | 4.39 | 2.47E-5 | 4.533 | 0.00559 | 0.00140 | 0.0316 |
| ssc05212:Pancreatic cancer | *EGFR, JAK1, TGFB3, PIK3R5, TGFA, IKBKB, RB1, AKT2, MAP2K1, TGFB1* | 10 | 3.38 | 2.66E-5 | 6.20 | 0.00603 | 0.00121 | 0.0341 |
| ssc04068:FoxO signaling pathway | *IKBKB, USP7, TGFB1, PRKAA2, PRKAG1, EGFR, PCK1, FOXO3, TGFB3, PIK3R5, AKT2, MAP2K1, CCND2* | 13 | 4.39 | 1.35E-4 | 3.82 | 0.0301 | 0.00508 | 0.172 |
| ssc04922:Glucagon signaling pathway | *CPT1B, CPT1A, PRKAA2, PHKG2, PRKAG1, PCK1, ACACB, PHKG1, ACACA, GYS1, AKT2* | 11 | 3.72 | 1.38E-4 | 4.52 | 0.0309 | 0.00448 | 0.177 |
| ssc04611:Platelet activation | *COL5A1, ITGB1, ORAI1, PIK3R5, AKT2, GUCY1A1, STIM1, NOS3, FYN, PRKCZ, PRKG1, VWF* | 12 | 4.05 | 4.49E-4 | 3.61 | 0.0969 | 0.0127 | 0.5738 |
| ssc04921:Oxytocin signaling pathway | *NFATC2, CACNB4, RYR1, CAMK1D, KCNJ5, CACNG1, GUCY1A1, NOS3, PRKAG1, PRKAA2, EGFR, PIK3R5, MAP2K1* | 13 | 4.39 | 7.04E-4 | 3.19 | 0.148 | 0.0176 | 0.897 |
| ssc05223:Non-small cell lung cancer | *EGFR, FOXO3, PIK3R5, TGFA, RB1, AKT2, MAP2K1* | 7 | 2.36 | 0.00174 | 5.36 | 0.327 | 0.0388 | 2.21 |
| ssc04920:Adipocytokine signaling pathway | *CPT1B, CPT1A, PRKAA2, PRKAG1, PCK1, ACACB, IKBKB, AKT2* | 8 | 2.70 | 0.00208 | 4.40 | 0.376 | 0.0420 | 2.63 |
| ssc00620:Pyruvate metabolism | *PC, PCK1, ACACB, MDH2, ACACA, ALDH2* | 6 | 2.03 | 0.00228 | 6.33 | 0.404 | 0.0422 | 2.88 |
| ssc04350:TGF-beta signaling pathway | *ACVR2A, ACVR1B, TGFB3, BMP7, BMPR1B, BMP6, BAMBI, TGFB1* | 8 | 2.70 | 0.00331 | 4.06 | 0.529 | 0.0562 | 4.15 |
| ssc04022:cGMP-PKG signaling pathway | *NFATC2, PIK3R5, ATP1A2, AKT2, GUCY1A1, ADRA1A, NOS3, ADRB2, MAP2K1, ATP1A1, PRKG1, VDAC1* | 12 | 4.05 | 0.00346 | 2.81 | 0.544 | 0.0546 | 4.33 |
| ssc04151:PI3K-Akt signaling pathway | *COL5A1, JAK1, FGF9, IKBKB, GYS1, NOS3, PPP2R2B, VWF, PRKAA2, PCK1, EGFR, FOXO3, ITGB1, PIK3R5, TNXB, AKT2, MAP2K1, CCND2* | 18 | 6.08 | 0.00378 | 2.16 | 0.577 | 0.0557 | 4.73 |
| ssc01212:Fatty acid metabolism | *CPT1B, CPT1A, HADHA, FASN, ACACA, FADS2* | 6 | 2.03 | 0.00542 | 5.21 | 0.709 | 0.0742 | 6.72 |
| ssc05211:Renal cell carcinoma | *TGFB3, PIK3R5, TGFA, AKT2, EPAS1, MAP2K1, TGFB1* | 7 | 2.36 | 0.00553 | 4.27 | 0.716 | 0.0714 | 6.85 |
| ssc04550:Signaling pathways regulating pluripotency of stem cells | *ACVR2A, BMI1, JAK1, ACVR1B, PIK3R5, BMPR1B, SOX2, AKT2, MAP2K1, WNT4* | 10 | 3.38 | 0.00577 | 3.00 | 0.731 | 0.0703 | 7.13 |
| ssc04917:Prolactin signaling pathway | *FOXO3, LHB, PIK3R5, AKT2, ELF5, MAP2K1, CCND2* | 7 | 2.365 | 0.00643 | 4.14 | 0.769 | 0.0741 | 7.92 |
| ssc00020:Citrate cycle (TCA cycle) | *PC, PCK1, MDH2, ACO2, ACLY* | 5 | 1.69 | 0.00664 | 6.51 | 0.780 | 0.0728 | 8.17 |
| ssc04380:Osteoclast differentiation | *CYBA, TYK2, JAK1, NFATC2, PIK3R5, IKBKB, AKT2, FYN, MAP2K1, TGFB1* | 10 | 3.38 | 0.00668 | 2.94 | 0.782 | 0.0699 | 8.22 |
| ssc04071:Sphingolipid signaling pathway | *CTSD, PIK3R5, S1PR2, AKT2, NOS3, FYN, PRKCZ, MAP2K1, PPP2R2B* | 9 | 3.04 | 0.00756 | 3.14 | 0.821 | 0.0753 | 9.25 |
| ssc05220:Chronic myeloid leukemia | *TGFB3, PIK3R5, IKBKB, RB1, AKT2, MAP2K1, TGFB1* | 7 | 2.36 | 0.00797 | 3.96 | 0.837 | 0.0759 | 9.73 |


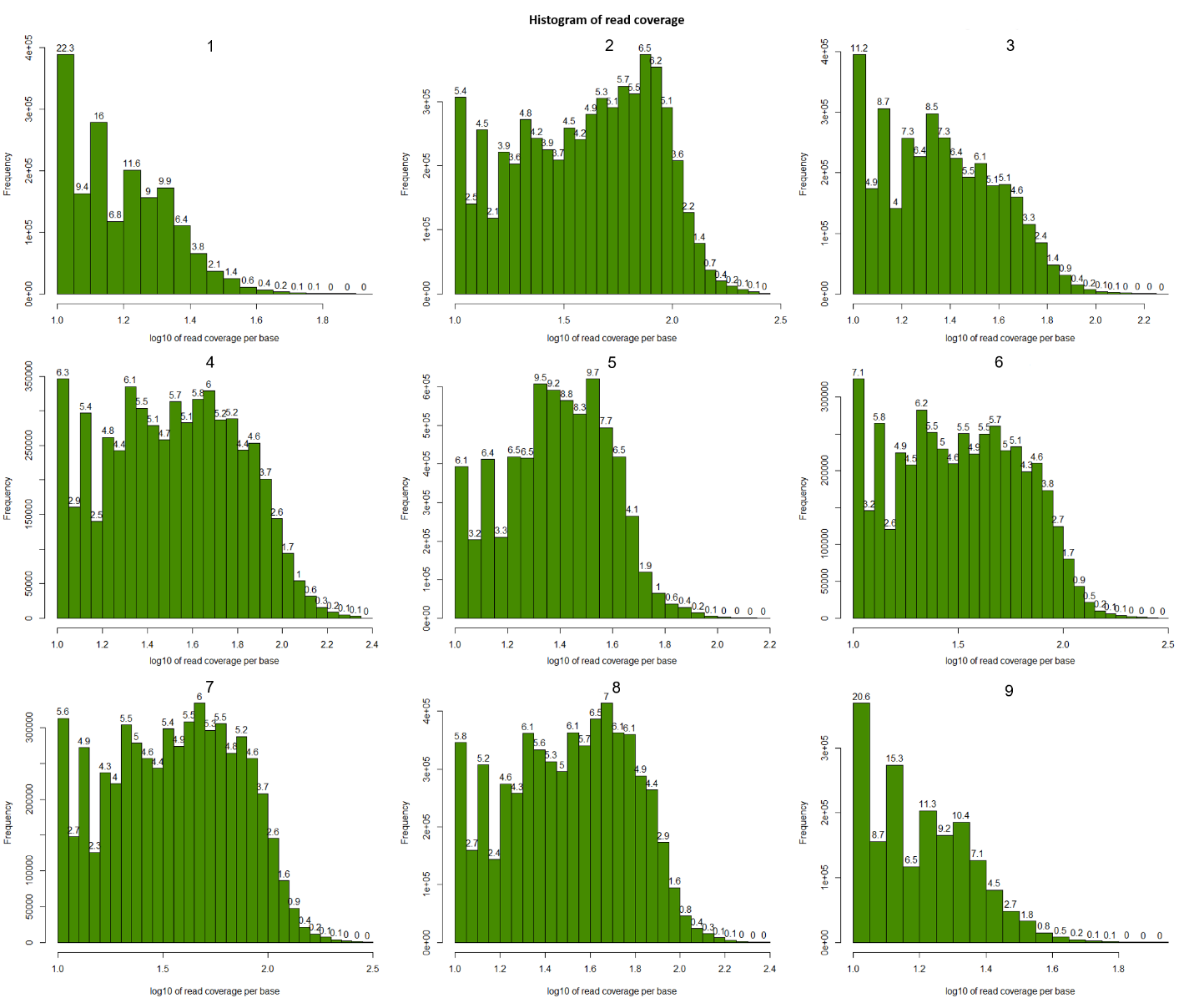


Supplementary figure 1. Histograms of log10 of read coverage per CpG site


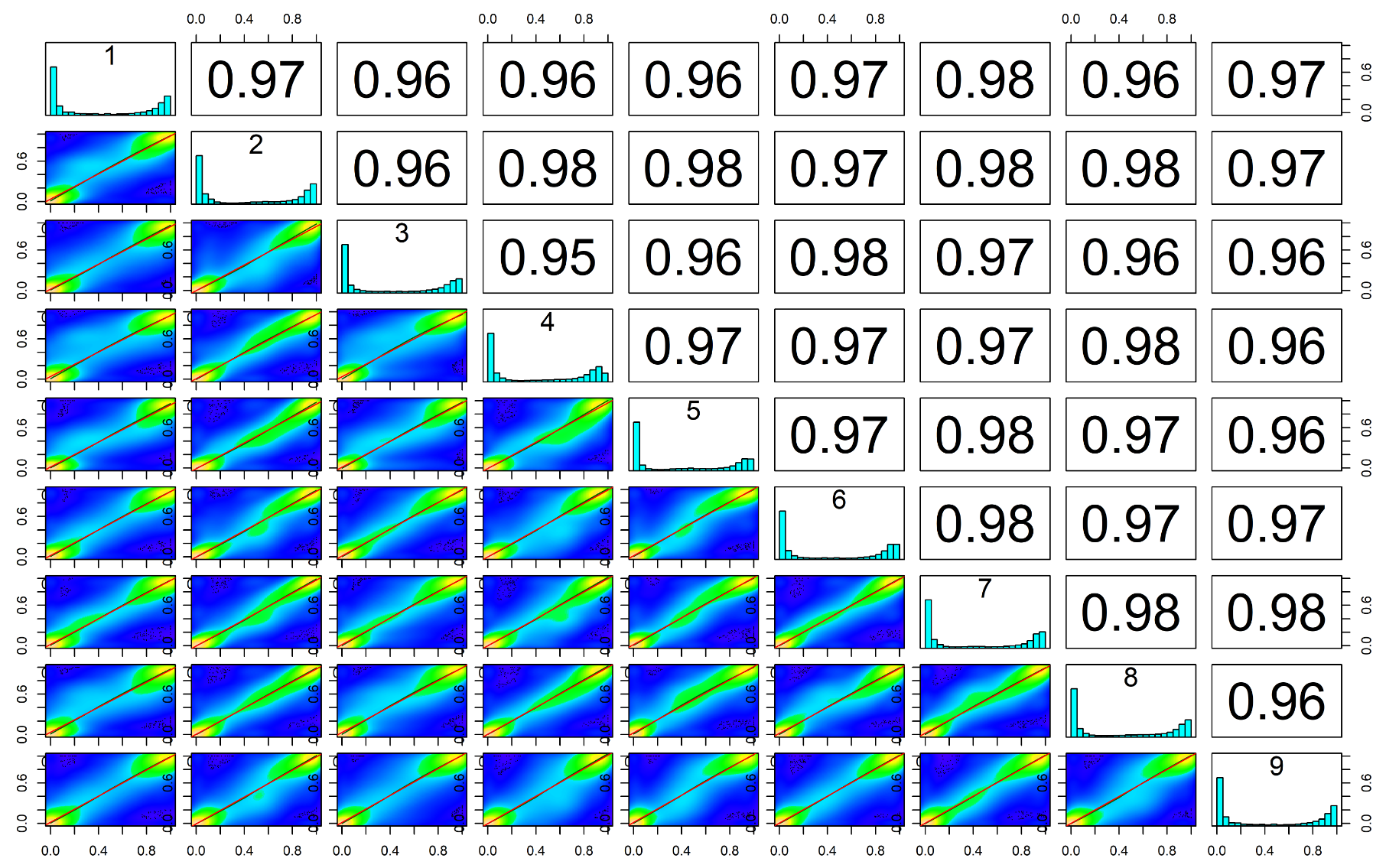


Supplementary figure 2. Correlation analysis of the global CpG methylation patterns among nine samples. Note: Colors in the scatter plot indicate the number of CpG sites with identical methylation pattern (methylated or non-methylated): yellow denotes many correlations, blue denotes lack of correlation and green denotes different methylation patterns. Numbers in the upper right side represent the pairwise Pearson's correlation scores. Histograms on the diagonal are methylation distribution per CpG site for each sample.


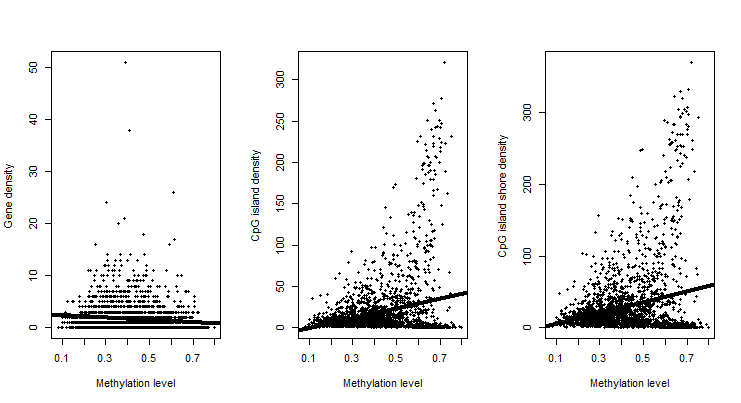


Supplementary figure 3. Regression of densities of genes, CpG islands and CpG island shores on methylation levels from one sample, all counted by 1 Mb windows.
